# Patient satisfaction with E-Oral Health care in rural and remote settings: a systematic review protocol

**DOI:** 10.1186/s13643-017-0550-3

**Published:** 2017-08-29

**Authors:** Elham Emami, Naomi Kadoch, Sara Homayounfar, Hermina Harnagea, Patrice Dupont, Nicolas Giraudeau, Rodrigo Mariño

**Affiliations:** 1 Department of Restorative Dentistry, Faculty of Dentistry, Université de Montréal, Montréal, Québec Canada; 2 School of Public Health, Université de Montréal, Montréal, Québec Canada; 3 Public Health Research Institute, Centre hospitalier de l’Université de Montréal, Université de Montréal, Montréal, Québec Canada; 4 Faculty of Dentistry, McGill University, Montréal, Québec Canada; 5 Faculty of Dentistry, Université de Montréal, Montréal, Canada; 6 Université de Montréal, Montréal, Canada; 7 Health Sciences Library, Université de Montréal, Montréal, Canada; 8 Faculty of Odontology, Université de Montpellier, Montpellier, France; 9 Melbourne Dental School, University of Melbourne, Melbourne, Australia

**Keywords:** E-Health, E-Oral Health, Teledentistry, Rural and remote communities, Patient satisfaction

## Abstract

**Background:**

Individuals living in rural and remote settings face oral health problems and access-to-care barriers due to the shortage of oral health care providers in these areas, geographic remoteness, lack of appropriate infrastructure and lower socio-economic status. E-Oral Health technology could mitigate these barriers by providing the delivery of some aspects of health care and exchange of information across geographic distances. This review will systematically evaluate the literature on patient satisfaction with received E-Oral Health care in rural and remote communities.

**Methods:**

This systematic review will include interventional and observational studies in which E-Oral Health technology is used as an intervention in rural and remote communities of any country worldwide. Conventional oral health care will be used as a comparator when provided. Patient satisfaction with received E-Oral Health care will be considered as a primary outcome for this review. Cochrane Central Register of Controlled Trials, MEDLINE, EMBASE and Global Health will be searched using a comprehensive search strategy. Two review authors will independently screen results to identify potentially eligible studies and independently extract the data from the included studies. A third author will resolve any discrepancies between reviewers. Two independent researchers will assess the risk of bias and the Grading of Recommendations Assessment, Development, and Evaluation.

**Discussion:**

The potential implications and benefits of E-Oral Health care can inform policymakers and health care professionals to take advantage of this technology to address health care challenges in these areas.

**Systematic review registration:**

PROSPERO CRD42016039942.

**Electronic supplementary material:**

The online version of this article (doi:10.1186/s13643-017-0550-3) contains supplementary material, which is available to authorized users.

## Background

Health care organizations deem health inequalities to be of paramount importance and constantly aim to ensure the equitable distribution of services [1]. Even so, disparities persist worldwide as a challenge for accessing health and oral health services, especially in rural and remote communities [2]. The problem of widened disparities in regard to access to oral health care services in rural and remote areas is recognized as being due to factors such as geographical isolation, limited availability and accessibility of dental professionals, population vulnerability, higher rates of poverty, socioeconomic deprivation, diminished public services, deficient infrastructure and lower rates of private dental insurance coverage [2–4]. This has led to lower dental care utilization, poorer oral health outcomes and dissatisfaction with oral health care in rural populations compared to urban populations [5, 6]. Furthermore, rural dentists, who do not have access to specialist opinions, may feel incompetent or make errors during their care decision-making or when providing complex treatments. Hence, professional incompetency such as inaccurate diagnosis may be harmful for patients or may lead to patient dissatisfaction with care and can create barriers in regard to optimal health care [2, 7–9].

Patient satisfaction is an important outcome measure for health care services and, as defined by Pascoe, is a patient’s response to a significant aspect of her/his experience of health care services [10, 11]. Patient’s satisfaction with health care includes various dimensions such as the technical quality of care, accessibility and availability of care, physical setting, financial issues and continuity of care. A global measure of satisfaction with received care may reflect several aspects of care, especially in the context of rural and remote settings; thus, it should be taken into account when addressing health disparities [11].

Technology is a major driving force of human civilization and has always been intertwined with human development [12]. The utilization of technology in providing and delivering health care has broadened globally [13]. In order to tackle health and oral health disparities, a paradigm shift is required in information and communication technologies in order to achieve greater emphasis on developments which benefit health [12]. However, in the same context, many solutions have been proposed in order to overcome these disparities, among which E-Health technology is experiencing the most rapid growth [13].

According to Eysenbach’s definition, “E-Health is an emerging field in the intersection of medical informatics, public health and business, referring to Health services and information delivered or enhanced through the Internet and other related technologies” [14]. E-Health is a broad term that encompasses not only technological development but also a way of global thinking and commitment to improving health care services worldwide, using information and communication technology [15, 16]. Telemedicine, m-health, telehealth and teledentistry, among others, fall under the umbrella term of E-Health. E-Health can help overcome the barriers of geographic distances that rural populations face through the delivery and exchange of health care information and specialists’ opinions across distances [17]. The first worldwide application of E-Oral Health technology was in fact designed to extend dental diagnosis and treatment in rural areas. The project was conducted in 1994 by the US military [16, 18]. Today, the use of E-Oral Health technology has been reported in dental education, preventive dentistry and oral medicine, among many other areas [19, 20]. The most common forms of telecommunication involve real-time consultations, store-and-forward consultations or a combination of both [15, 18, 20].

Our scoping searches of the most relevant databases showed that the original research on this topic is observational in nature and lacks the use of robust qualitative research methodology or mixed-methods studies. Furthermore, no systematic reviews have been carried out on the effect of E-Oral Health technology on patient satisfaction in rural and remote settings [18, 21–25]. Given the fact that a large number of E-Health strategic plans are being developed in rural and remote areas across the world, further investigation on this topic will support policy decision-making and planning for E-Oral Health programs, which will lead to the improvement of oral health and oral health care in rural and remote areas. Furthermore, a systematic review reporting on patients’ satisfaction with received E-Oral Health care will help the development of future research using qualitative methodology to explore patients’ experience with E-Oral Health care.

### Objectives

This systematic review aims to answer the following questions:

1. When compared with conventional oral health care, do E-Oral Health care interventions improve the satisfaction of patients in rural and remote settings with received oral health care?
2. Is the harmful effect of diagnostic errors made in E-Oral Health care interventions in patients in need of oral health care in rural and remote settings comparable to those in conventional oral health care?
3. To what extent does E-Oral Health care improve patient satisfaction with care in terms of reducing waiting time, number of visits, travel and the cost of care for patients in need of oral health care in rural and remote settings, when compared to conventional oral health care?

## Methods

### Protocol and registration

This systematic review protocol has been registered in PROSPERO (International Prospective Register of Systematic Reviews), under registration number CRD42016039942. The protocol has been carried out in accordance with the Preferred Reporting Items for Systematic Review and Meta-Analysis Protocols 2015 (PRISMA-P, Additional file 1) [26, 27].

### Eligibility criteria

Studies will be selected according to the following criteria:

#### Study design

Original research studies with a defined quantitative methodological approach (interventional or observational) including randomized clinical trials, quasi-experimental trials, longitudinal cohorts and cross-sectional surveys will be included in this review. Any case reports, position papers and reviews will be excluded from the review.

#### Participants

Participants of any age, sex, ethnicity, socioeconomic status, occupation and associated morbidities will be considered for this review. However, the review will consider only participants in need of oral health care. Oral health care will be defined as any type of oral health care including oral health care education, consultation, diagnosis and treatment.

#### Interventions

The review will consider as the intervention of interest the types of oral health care that are provided by E-Health technology. E-Health is defined here according to the definition provided by Eysenbach [14] and includes any type of E-Oral Health technology that could address the oral health needs of participants in terms of education, consultation, screening, diagnosis, treatment, support or any other type of application in the field of dental medicine [18]. No limitation in terms of the duration of the intervention and the type of stakeholders that are involved in the interventions will be imposed.

#### Comparators

Conventional oral health care will be defined as traditional approaches to oral health care having the same objectives as those defined under the intervention inclusion criteria, but without using E-Health technology. These will include patients’ education, consultation, disease screening, diagnosis, treatment and support or any other type of application in the field of dental medicine.

We expect that some observational studies will lack comparators. In this case, we will use historical knowledge for projecting the outcomes in regard to conventional oral health care [28].

#### Outcomes

The main outcome of the review will be patient satisfaction with received oral health care using self-reported measures, at any time after the intervention. The other primary outcome for the review will address undesirable consequences of the health care (E-Health or conventional). We will consider diagnostic error during care as an undesirable outcome, which will have a harmful effect on the care user.

The secondary outcome is the change in access to care and will be reported as a composite measure including waiting time, number of visits, travel and the cost of oral health care.

#### Timing

The eligible time point for the study selection will be immediately after the intervention. The length of the follow-up will not be considered for the selection of the studies since the primary outcome of interest (patient satisfaction with received oral health care) can be measured at any time after the intervention. Furthermore, since we include both interventional and observational studies (including cross-sectional), we expect that follow-up data may not be available. However, we will present a summary effect over all time points if the data are available.

#### Setting

Rural and remote communities will be considered as eligible settings for a study’s inclusion in the systematic review. There exist several definitions for rurality, and the choice of definition is based on the research questions [29]. In this review, the geographical aspect of rurality will be of interest rather than its social representations [30].

The dichotomous division of urban/rural will be used to define rurality [29] since variability in the rural zone is not within the context of review analysis. There will be no limits on the worldwide location and type of rural or remote communities. We will contact the studies’ authors if the information in the study in regard to setting is inadequate.

#### Language

For pragmatic reasons, only English and French language publications will be considered for full-text analysis in this systematic review. This will be considered as a limitation for the study.

### Information sources

An electronic literature search will be conducted in the following databases: Cochrane Central Register of Controlled Trials (The Cochrane Library, current issue), MEDLINE (OVID interface, 1946 onwards), EMBASE (OVID interface, 1974 onwards) and Global Health (OVID interface, 1973 onwards). The electronic literature search will be complemented by hand searching the list of references in the identified publications or relevant reviews. NICE Evidence and TRIP database will be searched for gray literature using subject keywords. Members of the E-Oral Health Network of the International Association of Dental Research will be contacted by email to identify any unpublished studies. Ongoing studies will not be considered in the review.

### Search strategy

A draft version of literature search strategies to be used in this work has been developed with the help of an expert librarian at Université de Montréal (PD) and a researcher with experience in the conduct of systematic reviews (EE) using medical subject headings (MeSH), EMTREEs and text words related to the field of the study. A draft of the MEDLINE search strategy can be found in Additional file 2. Once the MEDLINE search strategy is finalized, it will be adapted to the other databases using the proper syntax, subject headings and controlled vocabulary considering maximized sensitivity of the search. There will be no language restrictions in the search strategy to maximize the sensitivity and to identify the number of publications in other languages and to verify the existing risk of bias.

The electronic literature search will be complemented by hand searching the list of references in the identified publications or relevant reviews. NICE Evidence and TRIP database will be searched for gray literature using subject keywords. The clinical trial registry and PROSPERO will be searched for ongoing or recently completed studies and systematic reviews, respectively. The references contained in relevant studies will be checked for other relevant publications.

### Data management, selection and date collection process

The identified articles from search results will be transferred to EndNote software.

A screening tool will be developed according to inclusion and exclusion areas. The process of data selection and collection will be pilot tested in 10% of randomly selected included articles. Cohen’s kappa test will be used to assess the reviewers’ agreement on study eligibility [31].

Two independent reviewers will screen all retrieved titles and abstracts using the inclusion criteria. In case of incomplete information provided by the title and abstract, the full text will be used to determine a study’s eligibility to be included for the full analysis. In order to avoid overlapping data, publications related to the same study will be verified, and the most relevant report (according to study outcomes) will be selected for full review. The reviewers will not be blind to all content of the publications.

Any discrepancy between reviewers will be discussed and resolved through consensus. If an agreement cannot be obtained, the opinion of a third reviewer will be sought or the study authors will be contacted by email to obtain additional information.

### Data items

Two reviewers will independently extract the data from the full text of the included studies by adapting the review form from Effective Practice and Organization of Care (EPOC) Resources for review authors [32], as a data extraction method (Additional file 3). A meeting will be held for the data extraction and a pilot test will be conducted to get feedback from the reviewers and to ensure consistency across them. The extraction form will be modified if necessary to confirm its completeness.

The extracted information will include general information about publication such as sources of funding and possible conflicts of interest, authors, country, year of study publication, aim of the study, study design, sample size, participant characteristics (age, gender), target population, intervention description, type of E-Oral Health technology, conventional comparator, the outcome of interest, other outcomes, measurement instruments, the length of follow-up and the main results. In the case of missing information, an attempt will be made to contact the study authors.

### Outcomes and prioritization

As stated in the ‘Outcomes’ section, patient satisfaction with received oral health care is the main outcome of the review. This decision is based on the fact that patient satisfaction is among the outcomes that will lead to a higher level in the strength of related recommendations [33]. The undesirable primary outcome will be the diagnostic error during care. The secondary outcome is the change in access to care and includes defined composite measures: waiting time, number of visits, travel and the cost of oral health care.

### Risk of bias in individual studies

Two reviewers will independently assess the quality of the reports and the risk of bias. For the assessment of experimental studies, the Cochrane Collaboration tool for assessing the risk of bias will be used and will cover the following: randomization sequence generation, treatment allocation concealment, blinding, completeness of outcome data, selective outcome reporting and other sources of bias [34]. The assessment of observational studies will be performed using the ROBINS-I risk of bias assessment tool for non-randomized studies [35]. Disagreement will be resolved by consultation with a third reviewer.

### Data synthesis

The descriptive synthesis will be conducted in line with the guidance from the Centre for Reviews and Dissemination [36]. Text and tables will summarize and explain the characteristics of the findings in the included studies.

Where possible, the mean differences with 95% confidence interval (CI) will be calculated for continuous outcomes (patient satisfaction). Patients’ satisfaction change scores will be computed. Effect sizes (ES) will be calculated to compare the results across studies. Effects will be expressed as standard mean differences (SMD).

For dichotomous outcomes (the presence of diagnostic errors, access to care), odds ratio with 95% CI will be reported. Since access to care is a composite outcome, the defined items will be combined before dichotomization. The summary likelihood ratios will be calculated.

Where appropriate (availability of two or more studies with similar study design, measures and outcomes), meta-analyses will be carried out using a random-effects model [37–39]. This approach is preferable to a fixed model since it accounts for inter-study variation and provides a more conservative estimate. The unit of analysis will be the patient, and the outcome variables will be grouped to enable the meta-analyses. When comparisons are made between pooled standardized mean differences (for continuous variables), statistical differences will be assessed using a *Z* test and *p* < 0.05 will be considered significant [38, 39]. For dichotomous variables, the Mantel-Haenszel method will be used to pool the data. Pool effect sizes as well as their 95% confidence limits will be reported. The Cochrane *Q* test and *I* ^2^ statistic will be used to test heterogeneity [40, 41]. Subgroup analyses (patient characteristics, type of e-tool, degree of rurality) will be conducted to identify the sources of heterogeneity across the studies.

If the selected publications include qualitative data, a thematic analysis approach [23, 42] will be included in the data synthesis. Furthermore, if the studies provided insufficient quantitative data (missing data, unpublished outcome), we will contact authors for additional information. If the missing data could not be rectified by author contact via email, we will use narrative approaches to describe the major findings.

### Meta-biases

To evaluate the risk of selective reporting, the registered protocols of trials will be checked. Funnel plots will be used to identify potential publication bias [43]. Tests for funnel plot asymmetry will be considered if the number of studies included in the meta-analysis is more than 10 [38].

### Confidence in cumulative evidence

We will evaluate the level of evidence of all studies according to the Oxford Level of Evidence [44]. The Grading of Recommendations Assessment, Development, and Evaluation (GRADE) approach will be used to summarize the evidence [45]. We will use a table to summarize the overall confidence in the evidence as either high, moderate, low or very low.

### Differences between the protocol and the review

Although it is important that protocols of systematic review be available to avoid selective reporting, many such protocols are later modified because of issues that have not been anticipated at the protocol stage [46].

If for some reasons, we are not able to follow the protocol plan, the deviations will be described in the final review. This will include any important changes in the methods of the review.

## Discussion

The recent development of E-Health technologies and their integration and implementation in primary oral health care by interdisciplinary teams has the potential to address the dental needs of individuals in remote and rural communities, to satisfy them and to alleviate the burden of access to care.

To globally establish E-Oral Health care, specifically in rural and remote areas all around the world, more research is needed to provide evidence of its benefits, especially from end-users’ perspectives. The findings from this review will be used to address the lack of knowledge on the fragmentation of rural dental care, where access to dental health care is less available. The findings also have the potential to empower the isolated dental workforce working in rural and remote zones across the world.

This study review is limited due to its narrow inclusion criteria in regard to language and inclusion of various study designs, and caution should be taken when interpreting the results.

## Additional files

Additional file 1: PRISMA-P+checklist. (DOCX 42 kb)

Additional file 2: MEDLINE search strategy. (DOCX 16 kb)

Additional file 3: Data extraction form draft. (DOCX 47 kb)
