# Supplementary material for: Patient satisfaction with E-Oral Health care in rural and remote settings: a systematic review protocol
Source: Syst Rev. 2017 Aug 29;6:174. doi: 10.1186/s13643-017-0550-3 (PMC5576324; doi:10.1186/s13643-017-0550-3)
Supplement: Supplementary file 1 — PRISMA-P+checklist. (DOCX 42 kb) [file 13643_2017_550_MOESM1_ESM.docx]

| Database(s): All Ovid MEDLINE(R) 1946 to Present |  |
| --- | --- |
| Search Strategy: |  |
| **#** | **Searches** |
| 1 | (mobile dentistry or teledentistry or tele-dentistry or edentistry or mdentistry or e-dentistry or m-dentistry or dental telecommunication).mp. |
| 2 | exp telemedicine/ or exp remote consultation/ or exp Videoconferencing/ or (mobile health or telehealth or telemedicine or tele-health or tele-medicine or ehealth or mhealth or e-health or m-health or teleconsult* or telepatholog* or telemonitor* or videoconferenc* or medical telecommunication or m-patient* or mpatient*OR remote consult* or tele-consult* or tele-patholog* or tele-monitor* or video-conferenc* or medical tele-communication).mp. |
| 3 | Rural Health/ or exp Rural Health Services/ or Rural Population/ or Hospitals, Rural/ or (rural or Remote or country area or country areas or countryside* or country-side or country-sides or aboriginal or native* or tribe* or indigenous).mp. |
| 4 | 1 and 3 |
| 5 | exp dentistry/ or exp oral health/ or exp mouth diseases/ or exp Dental Care/ or exp dental caries/ or exp education, dental/ or exp diagnosis, oral/ or (oral health care or dental or dentist* or endodonti* or oral medicine or oral or orthodonti* or periodonti* or prosthodonti* or tooth or teeth or mouth). mp. |
| 6 | 2 and 3 and 5 |
| 7 | 6 not 4 |
| 8 | ((exp Delivery of Health Care/ or exp Health Services Accessibility/ or exp Costs/) and Cost Analysis/) or exp diagnosis/ or exp patient satisfaction/ or patient acceptance of health care/ or waiting lists/ or efficiency, organizational/ or exp office visits/ or (access* or patient satisfaction or cost* or health care cost or waiting time or effectiveness or efficiency).mp. |
| 9 | 4 and 8 |
| 10 | 7 and 8 |

**Additional file 1: MEDLINE Search Strategy**
