# Supplementary material for: Patient satisfaction with E-Oral Health care in rural and remote settings: a systematic review protocol
Source: Syst Rev. 2017 Aug 29;6:174. doi: 10.1186/s13643-017-0550-3 (PMC5576324; doi:10.1186/s13643-017-0550-3)
Supplement: Supplementary file 3 — Data extraction form draft. (DOCX 47 kb) [file 13643_2017_550_MOESM3_ESM.docx]

# Data collection form adapted from

*Effective Practice and Organisation of Care (EPOC). Data collection form. EPOC Resources for review authors. Oslo: Norwegian Knowledge Centre for the Health Services; 2013*

Reviewer: ………………………………...

Date of review: …………………………

| **Review title** |
| --- |
| **Patient Satisfaction with e-Oral Health Care in Rural and Remote Settings: A Systematic Review Protocol** |

##

| **Study identification #**  ***Study Source :*** |
| --- |
| **Study title :** |

## General Information

| **General information** | | | |
| --- | --- | --- | --- |
| 1. Author | |  |  |
| 1. Published Journal | |  |  |
| 1. Year of the publication | |  |  |
| 1. Discipline (Field of application) | |  |  |
| 1. Year of the study | |  |  |
| 1. Country of the study | |  |  |
| 1. Sources of funding | |  |  |
| 1. Ethical approval | |  |  |
| 1. Possible conflict of interest | | Yes/No/ |  |
| 1. Contact with author | | Yes/No/date/reason |  |
| 1. Notes: |  | |  |

## Study eligibility

| **Study Characteristics** | | | **Review Inclusion Criteria** | **Yes/ No / N/A** | **Location in text**  *(pg & ¶/fig/table)* |
| --- | --- | --- | --- | --- | --- |
| 1. Type of the publication | | |  |  |  |
| 1. Study design | | |  |  |  |
| 1. Language | | |  |  |  |
| 1. Description of the intervention | | |  |  |  |
| 1. Description of the comparator | | |  |  |  |
| 1. Type of the e-health technology | | |  |  |  |
| 1. Target population | | |  |  |  |
| 1. Setting | | |  |  |  |
| 1. Timing | | |  |  |  |
| 1. Decision | | **Included [ ] / excluded [ ]** | | | |
| 1. Reason for exclusion | | |  | | |
| 1. Notes: |  | | | | |

**DO NOT PROCEED IF STUDY EXCLUDED FROM REVIEW**

## Methods

|  | | **Descriptions as stated in report/paper** | | | **Location in text**  *(pg & ¶/fig/table)* |
| --- | --- | --- | --- | --- | --- |
| 1. Total sample size | |  | | |  |
| 1. Number of participants by gender | | Female: Male: | | |  |
| 1. Aim of study | | Aim:  Not clearly defined [ ] | | |  |
| 1. Start date   *(dd-mm-yyyy)* | |  | | |  |
| 1. End date   *(dd-mm-yyyy)* | |  | | |  |
| 1. How was the participants defined? | |  | By gender  By age | Yes [ ] No [ ]  Define:  Mean ± SD,  median  (min-max yrs) |  |
|  |  |  | By Oral condition | Yes [ ] No [ ] |  |
|  |  |  |  | Mixed diseases and conditions:  Yes [ ] No [ ] |  |
|  |  |  |  | If yes which: |  |
|  |  |  | Type of rural setting |  |  |
|  |  |  | Other | |  |
| 1. Intervention | |  | | |  |
| 1. Intervention field of application | |  | | |  |
| 1. Comparator | |  | | |  |
| 1. Comparator field of application | |  | | |  |
| 1. The type of e-health technology | |  | | |  |
| 1. Primary outcome(s) | | 1a.  1b.  1c. | | |  |
| 1. Secondary outcome(s) | | 2a.  2b.  2c. | | |  |
| 1. Measurement instrument for primary outcome (s) | | 1a.  1b.  1c. | | |  |
| 1. Measurement instrument for secondary outcome (s) | | 2a.  2b.  2c. | | |  |
| 1. Notes: |  | | | | |

## Results

|  | | **Description as stated in report/paper** | | **Location in text**  *(pg & ¶/fig/table)* |
| --- | --- | --- | --- | --- |
| 1. Unit of analysis | |  | |  |
| 1. Time point   *(specify whether from start or end of intervention)* | |  | |  |
| 1. Primary outcome Results   1a.  1b.  1c. | | Intervention | Comparison |  |
| 1. Secondary outcome Results   2a.  2b.  2c. | | Intervention | Comparison |  |
| 1. No. missing participants and reasons | | Intervention | Comparison |  |
| 1. Any other results reported | |  | |  |
| 1. Statistical methods used | |  | |  |
| 1. Notes: |  | | | |
